# Impact of melphalan day -1 vs day -2 on outcomes after autologous stem cell transplant for multiple myeloma

**DOI:** 10.3389/fimmu.2024.1310752

**Published:** 2024-03-05

**Authors:** Aimee Merino, Ryan Shanley, Faridullah Rashid, Jenna Langer, Michelle Dolan, Sarah Tu, Najla El Jurdi, John Rogosheske, Kirollos Hanna, Todd DeFor, Murali Janakiram, Daniel Weisdorf

**Affiliations:** ^1^ Blood and Marrow Transplant Program, Department of Medicine, University of Minnesota, Minneapolis, MN, United States; ^2^ Department of Medicine, University of Minnesota, Minneapolis, MN, United States; ^3^ Department of Laboratory Medicine and Pathology, University of Minnesota, Minneapolis, MN, United States; ^4^ Department of Medicine, City of Hope, Duarte, CA, United States

**Keywords:** melphalan, autologous stem cell transplant, myeloma, engraftment, rehospitalization

## Abstract

**Background:**

Melphalan is the most common conditioning regimen used prior to autologous stem cell transplant (ASCT); however, there are varying data on optimal melphalan timing prior to transplant for best safety and efficacy. Historically, ASCT conditioning consisted of melphalan 200 mg/m^2^ on day 2 (D-2) (48 h prior to ASCT), but many institutions have since adopted a melphalan protocol with administration on day 1 (D-1) (24 h prior to SCT) or split dosing over the 2 days. The optimal timing of melphalan has yet to be determined.

**Methods:**

In this single-center retrospective study, we analyzed transplant outcomes for patients between March 2011 and September 2020 admitted for high-dose, single-agent melphalan 200 mg/m^2^ on D-1 vs. D-2. The primary outcomes were time to neutrophil and platelet engraftment. Secondary outcomes include incidence of hospital readmission within 30 days, 2-year progression-free survival, and 2-year overall survival.

**Results:**

A total of 366 patients were studied (D-2 n = 269 and D-1 n = 97). The incidence of high-risk cytogenetics was similar between the two groups (37% vs. 40%). Median days to absolute neutrophil count engraftment was similar at 11 days in the D-2 and D-1 cohort (n = 269, range 0–14, IQR 11–11 vs. n = 97, range 0–14, IQR 11–12). Median days to platelet engraftment >20,000/mcL was 18 days for D-2 melphalan (range: 0–28, IQR 17–20) versus 19 days for D-1 melphalan (range: 0–32, IQR 17–21). Overall survival at 2 years post-transplant was similar in both cohorts (94%; p = 0.76), and PFS was 70% in D-2 compared with 78% in D-1 (p = 0.15). In a multivariable model including age and performance status, hospital readmission within 30 days of transplant was higher in the D-1 cohort (odds ratio 1.9; p = 0.01).

**Conclusion:**

This study demonstrates similar neutrophil and platelet engraftment in D-1 and D-2 melphalan cohorts with similar 2-year PFS and OS. Either D-2 or D-1 melphalan dosing schedule is safe and effective.

## Introduction

Autologous stem cell transplantation (ASCT) after induction therapy remains the standard of care for transplant-eligible multiple myeloma (MM) patients. Even in the era of novel therapeutic agents, randomized studies continue to support the use of ASCT for consolidation therapy of MM (1–3). High-dose melphalan is nearly universally used as conditioning prior to ASCT owing to its established efficacy (4). Melphalan, an alkylating agent, is directly cytoreductive as well as immunomodulatory. Cytokine release after melphalan augments T-cell activity (5) and may promote natural killer (NK) cell activation (6) potentially limiting risks of relapse.

Several melphalan-conditioning regimens have been used including split dosing over 2 days or single-day melphalan on D-1 or D-2 prior to stem cell administration. D-2 dosing was historically used to avoid potential effects of residual melphalan on the stem cell graft. Two retrospective studies of MM patients undergoing ASCT observed no difference in time to platelet and neutrophil engraftment between D-1 and D-2 melphalan dosing (7–9), whereas a study of 367 patients showed delays in both platelet and neutrophil engraftment with D-1 melphalan (10).

A variation of melphalan dosing gives the 200-mg/m^2^ dose over 2 days. A study comparing melphalan given in 1 day (D-2) or over a 2-day period (days −3 through −2) saw no differences in platelet or neutrophil engraftment, progression-free survival (PFS), or overall survival (OS). More grade 3 or higher mucositis was seen in patients receiving melphalan over 2 days (11). A prospective study compared melphalan given in 1 day versus over 2 days and confirmed similar PFS, OS, and time to platelet engraftment. In contrast, neutrophil engraftment was significantly delayed in patients who got melphalan in 1 day vs. 2 days, although a larger stem cell dose was infused in the 2-day group (12).

We previously administered melphalan 200 mg/m^2^ day-2 (D-2) (48 h prior to ASCT) and switched to 200 mg/m^2^ on day-1 (D-1) (24 h prior to ASCT) to reduce hospital stay. All patients were planned for day +1 discharge. In this study, we compared engraftment outcomes, incidence of hospital readmission within 30 days of transplant, and progression-free survival (PFS) and overall survival (OS) between melphalan 200 mg/m^2^ given on D-2 versus D-1. Since PFS is highly influenced by cytogenetic risk, we collected cytogenetic data from bone marrow biopsies performed at diagnosis and graded risk using the Mayo Stratification for Myeloma and Risk-Adapted Therapy (mSMART) guidelines (13).

## Methods

A total of 366 multiple myeloma patients who underwent inpatient autologous transplant were included in this single-center retrospective analysis from March 2011 to September 2020. Each patient was admitted to the hospital for high-dose single-agent melphalan conditioning at a dose of 200 mg/m^2^. We excluded 26 patients who received melphalan at <200 mg/m^2^; 3 patients who received bone marrow grafts, and 12 patients with missing data. Melphalan dose received on D-1 (N = 97) (November 2017–September 2020) was given within 24 h prior to ASCT and melphalan on D-2 (N = 269) at 48 h prior to ASCT (March 2011–October 2017).

The primary endpoints were time to neutrophil and platelet engraftment. Engraftment was defined as the first of 3 days of absolute neutrophil count > 0.5 × 10^9^/L and the first of three consecutive platelet counts >20,000/mcL in the absence of platelet transfusions for 7 days. Secondary endpoints included incidence of readmission within 30 days from transplant starting from D-0, and PFS and OS at 2 years.

Inclusion criteria included clinically and pathologically confirmed MM in adults aged 18–75 years who received melphalan at 200 mg/m^2^. For hospital readmission as the end point, we also excluded patients who were continuously admitted from melphalan administration to engraftment or who were transplanted prior to June 2012 (n = 46) because of an institutional change from inpatient to planned outpatient transplantation (discharge D+1) at that time. No significant differences were seen in the demographic characteristics described in **Table 1**.

**Table 1:** Demographics and transplant characteristics.

Factors	Day receiving melphalan		
	Day −2	Day −1	p
**N**	269	97	
Age at transplant			0.03
Median (range), (IQR)	60 (33–76), (54–66)	64 (27–75), (58–68)	
Karnofsky Score at admission			0.12
60–80	69 (26%)	33 (34%)	
90–100	200 (74%)	64 (66%)	
**CMV serostatus:** positive	151 (56%)	52 (54%)	0.67
Comorbidity (HCT-CI)			0.64
Low risk (0)	102 (38%)	33 (34%)	
Intermediate risk (1–2)	99 (37%)	35 (36%)	
High risk (3+)	68 (25%)	29 (30%)	
Disease status at transplant			0.03
CR or sCR	49 (18%)	27 (28%)	
VGPR	120 (45%)	30 (31%)	
PR or SD	100 (37%)	40 (41%)	
Cytogenetic risk			0.72
High risk	99 (37%)	39 (40%)	
Standard risk	155 (58%)	56 (58%)	
Mobilization			
Cyclophosphamide	142 (53%)	37 (38%)	0.01
Plerixafor	72 (27%)	35 (36%)	0.08
CD34+ cell dose (×10^6^/kg)			<0.01
Median (range), (IQR)	5.9 (2.2–27.6), (4.1–8.5)	4.6 (2.6–21.6), (3.8–6)	
Months from diagnosis to transplant			0.02
Median (range), (IQR)	8 (3–146), (6–11)	7 (4–32), (5–9)	

IQR, interquartile range; CMV, cytomegalovirus; HCT-CI, hematopoietic cell transplantation-specific comorbidity index; sCR, stringent complete response; CR, complete response; VGPR, very good partial response; PR, partial response; SD, stable disease. Mobilization compares cyclophosphamide priming yes versus no (reference) and plerixafor added for graft mobilization yes versus no (reference).

Days to platelet and neutrophil engraftment were compared using the Wilcoxon rank-sum test (all patients engrafted thus none were censored). In order to assess the independent effect of melphalan day on engraftment, non-inflated negative binomial regression models were used (14). Predefined factors that were included as potential confounding variables were age (continuous per decade), transplant comorbidity risk (low risk versus intermediate risk versus high risk), CD34^+^ stem cell count (log continuous), and cyclophosphamide (CTX) priming for stem cell mobilization (yes versus no). The regression models produce incidence rate ratios (IRR) which can be interpreted as the increase (if greater than 1.0) or decrease (if less than 1.0) in the number of days until engraftment.

Secondary endpoints of OS and PFS were estimated by the Kaplan–Meier method and compared using the log-rank test (15). Multivariable Cox regression models were used to assess the independent effect on PFS of age (continuous per decade), cytogenetic risk, and disease status (complete response (CR) versus very good partial response (VGPR) versus partial response/stable disease (PR/SD) as predefined potential confounders (16). Response criteria were graded according to the International Myeloma Working Group definitions. Five patients who received a planned tandem transplant were excluded from OS and PFS analyses. A multivariable logistic regression model was used to compare readmission within 30 days of transplant by melphalan day, adjusted for age and KPS (90–100 vs. 60–80). The total number of hospital days and the number of visits in the bone marrow transplant clinic between day 0 and day 45 were compared between the groups.

All reported *p*-values were two-sided, and analyses were performed using SAS 9.4 (SAS Institute, Inc., Cary, NC) and R version 3.6.2.

## Results

A total of 366 patients were included in the analysis (D-2 melphalan n = 269, D-1 melphalan n = 97). Baseline characteristics that differed significantly between the cohorts were age, disease status at transplant, mobilization regimen, and time from diagnosis to transplant (**Table 1**). In the D-1 cohort, the median age was 64 years (range 27–75) and the median age in the D-2 group was 60 years (range 33–76) (*p* = 0.03). D-2 melphalan had a higher proportion receiving cyclophosphamide mobilization (53% vs. 38%, p = 0.01) and slightly lower proportion of plerixafor (27% vs. 36%, p = 0.08) for mobilization compared with the D-1 patients. There was a longer time between diagnosis and transplant in the D-2 group (median 8 vs. 7 months *p* = 0.02) that was partially explained by the higher use of cyclophosphamide mobilization in that group. Disease status also differed with a lower proportion of patients in CR in the D-2 group (18% vs. 28%) and a higher proportion in VGPR (45% vs. 31% *p* = 0.03).

**Figure 1:**
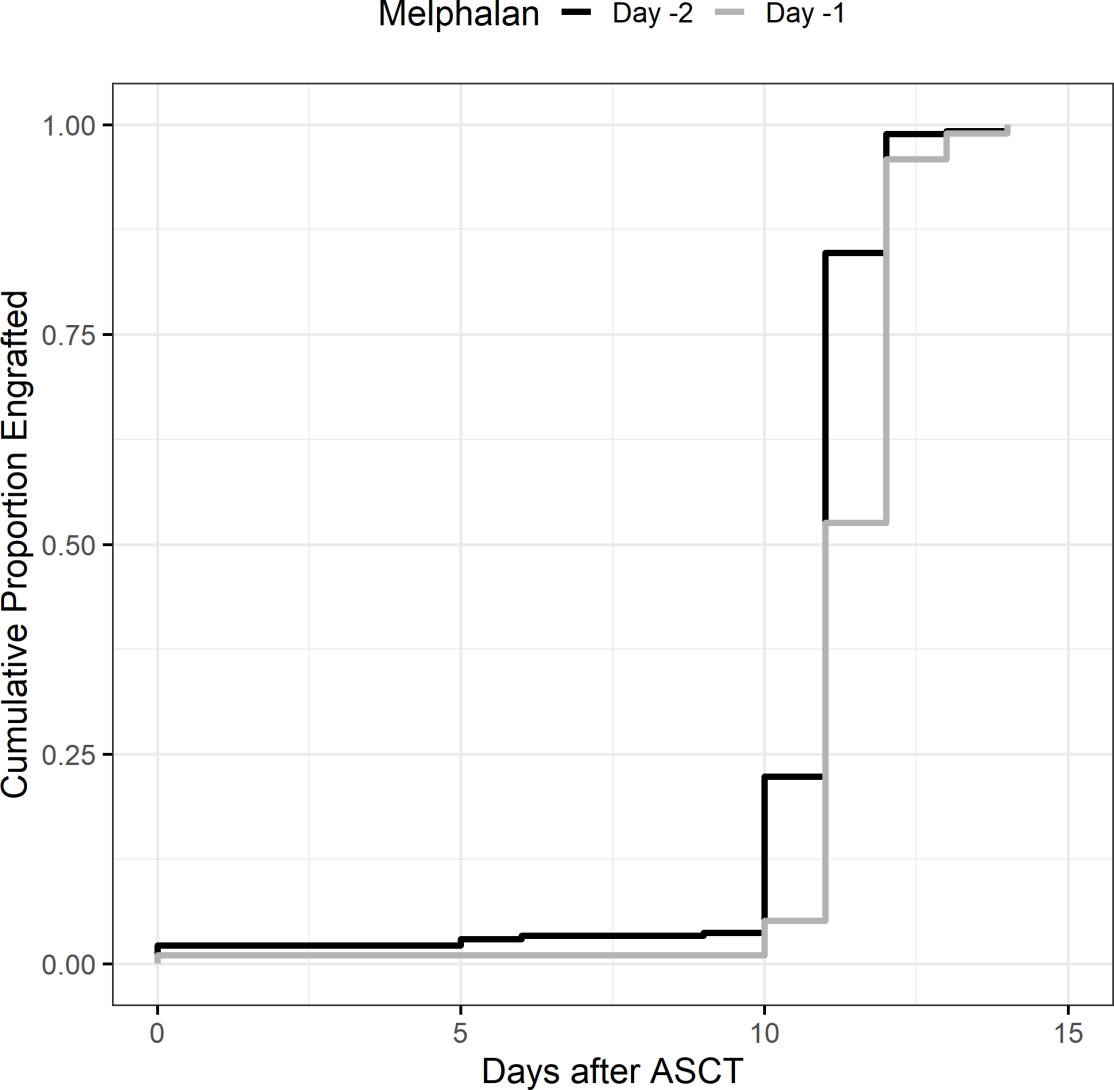
Neutrophil engraftment. Neutrophil engraftment was considered the first of 3 days of absolute neutrophil count >0.5 × 10^9^/L.

There were no engraftment failures in either cohort. Median days to absolute neutrophil count (ANC) engraftment was similar between groups at 11 days (range 0–14, IQR 11–11) in the D-2 cohort and 11 days in the D-1 cohort (range: 0–14, IQR 11–12) (**Table 2**), and the incidence rate ratio (IRR) of engraftment was 1.06 (95% CI 0.99–1.14, p = 0.12) between the D-2 and D-1 cohorts (**Figure 1**). Age, chemotherapy priming, hematopoietic cell transplantation comorbidity index (HCT-CI), and CD34^+^ cell dose were not associated with earlier engraftment (**Table 2**).

**Table 2A:** Days to neutrophil engraftment.

Factor		N	Median(range), (IQR)	Proportion engrafting
Overall		366	11 (0–14), (11–11)	100%
Melphalan day	−2	269	11 (0–14), (11–11)	100%
	−1	97	11 (0–14), (11–12)	100%

**Table 2B:** Negative binomial regression on the days to ANC engraftment.

Factor	N	Incidence rate ratio (IRR) (95% C.I.)	p
Melphalan day			
−2	269	1.0	
−1	97	1.06 (0.99–1.14)	0.12
**Age (/decade)**		1.01 (0.98–1.05)	0.50
Cyclophosphamide priming			
No	187	1.0 (reference)	
Yes	179	0.96 (0.89–1.03)	0.22
**Log CD34+ cell dose (×10^6^/kg)**		1.01 (0.94–1.08)	0.81
**Comorbidity (HCT-CI)**			0.94
Low risk	135	1.0 (reference)	
Intermediate risk	134	1.01 (0.94–1.09)	
High risk	97	1.01 (0.93–1.10)	

ANC engraftment was considered the first of 3 days of absolute neutrophil count >0.5 × 10^9^/L. The incidence rate ratio describes whether mean days to ANC engraftment was larger (IRR > 1.0) or smaller (IRR < 1.0) compared with the reference group. A patient who received melphalan on day −1 was expected to have 6% more days until ANC engraftment compared with a patient who received melphalan on day −2 and had the same age, priming, CD34+ cell dose, and HCT-CI.

**Figure 2:**
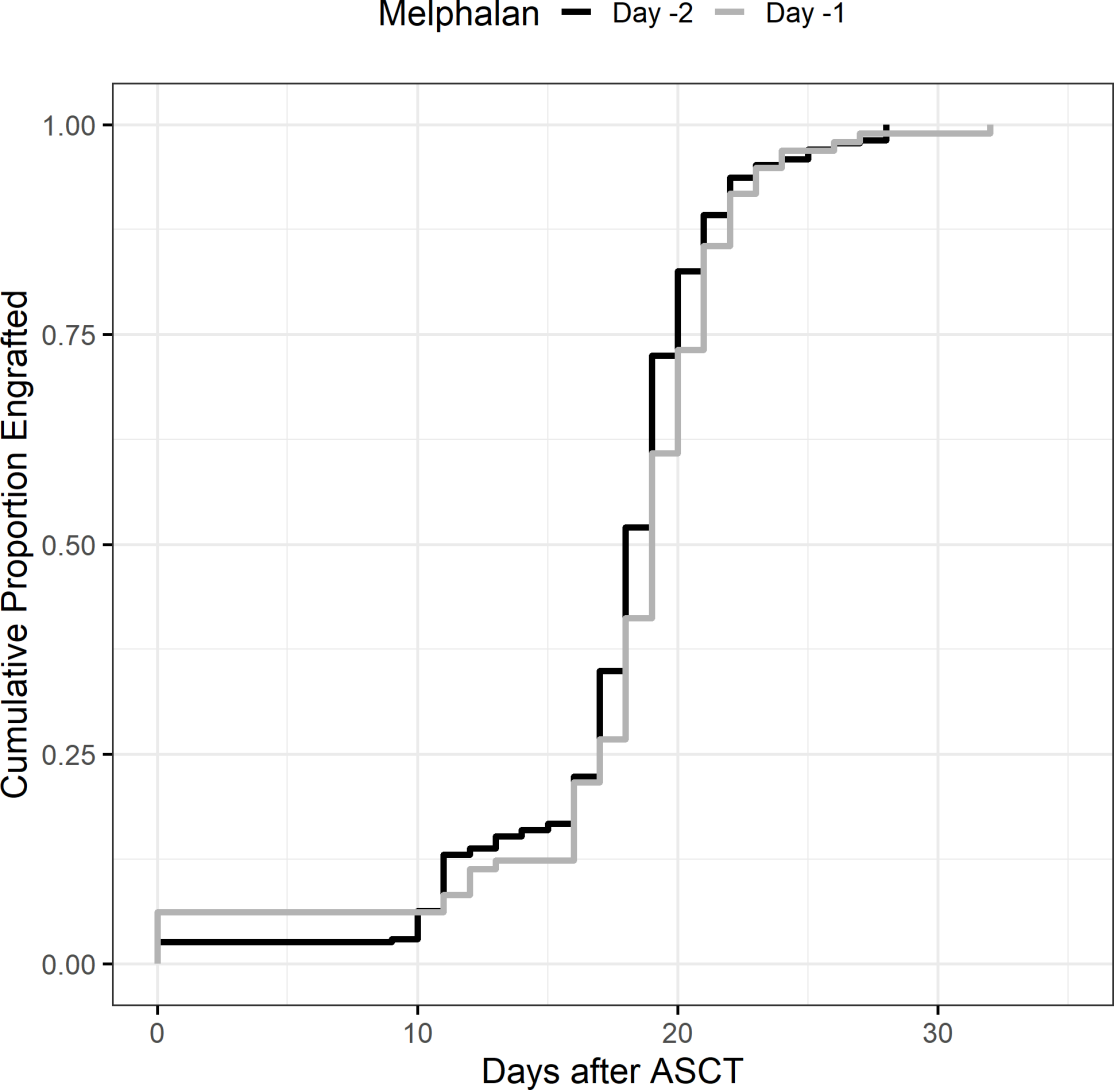
Platelet engraftment >20k.

Median days to platelet count over 20,000/mcL was reached at 18 days at D-2 (range: 0–28, IQR 17–20) versus 19 days for D-1 melphalan (range 0–32, IQR 17–21) (**Figure 2**). After adjusting for covariates including age, chemotherapy priming, HCT-CI index, and CD34^+^ cell dose, only CD34^+^ cell dose was associated with an earlier engraftment (IRR −0.86 p < 0.01) (**Table 3**).

**Table 3A:** Days to platelet engraftment (20,000/mcL).

Factor		N	Median(range), (IQR)	Proportion engrafting
Overall		366	19 (0–32),(17–20)	100%
Melphalan day	−2	269	18 (0–28), (17–20)	100%
	−1	97	19 (0–32), (17–21)	100%

**Table 3B:** Negative binomial regression on the days to platelet engraftment.

Factor	N	Incidence rate ratio (IRR) (95% C.I.)	p
Melphalan day			
−2	269	1.0	
−1	97	0.98 (0.92–1.05)	0.55
**Age (/decade)**		1.03 (1.00–1.07)	0.09
Cyclophosphamide priming			
No	187	1.0 (reference)	
Yes	179	0.98 (0.92–1.04)	0.46
**Log CD34+ cell dose (×10^6^/kg)**		0.86 (0.81–0.92)	<0.01
**Comorbidity (HCT-CI)**			0.99
Low risk	135	1.0 (reference)	
Intermediate risk	134	1.00 (0.93–1.07)	
High risk	97	1.02 (0.93–1.08)	

**Figure 3:**
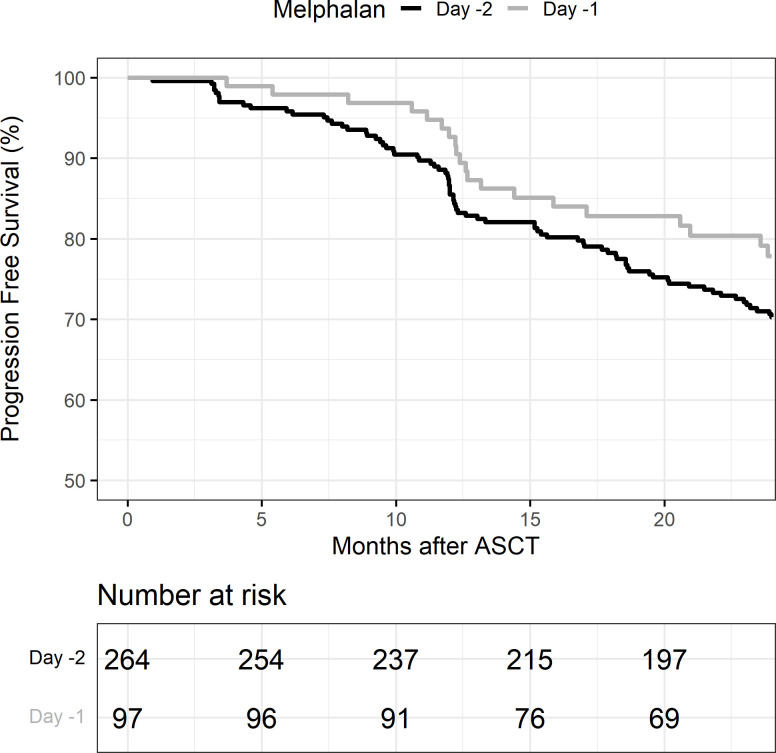
Progression-free survival (PFS) in melphalan D-2 vs. D-1. ASCT, autologous stem cell transplant.

OS at 2 years post-transplant was similar at 94% (95% CI: 90%–96%) in the D-2 cohort and 94% (95% CI: 84%–98%) in the D-1 cohort (p = 0.76) (**Table 4**). PFS at 2 years was also similar in the D-2 cohort at 70% (95% CI: 64%–75%) compared with 78% (95% CI: 68%–85%) in the D-1 cohort (*p* = 0.15) (**Figure 3**). There was one non-relapse death in the D-1 cohort, none in D-2. Multivariable Cox regression for PFS with age, cytogenetic risk, and disease status at transplant was performed with D-2 as the reference. Compared with D-2, the D-1 group showed a hazard ratio of 0.71 (95% CI 0.43–1.17, *p* = 0.18). High-risk cytogenetics had a significant adverse effect on PFS with a hazard ratio of 1.64 (95% CI 1.09–2.49; p = 0.02) compared with standard risk cytogenetics (reference group) or patients with unknown risk (HR = 1.54; 95% CI 0.65–3.63) (*p* = 0.02).

**Table 4A:** Progression-free and overall survival at 2 years post-transplant.

Progression-free survival				
Melphalan day	N	2-year PFS	95 CI%	p-value
−2	264	70%	64%–75%	0.15
−1	97	78%	68%–85%	

**Table 4B:** Progression free survival at 2-years post transplant.

Multivariable Cox regression model of PFS			
Factor	N	Hazard ratio (95% CI)	p
Melphalan day			
−2	264	1.0 (reference)	
−1	97	0.71 (0.43, 1.17)	0.18
**Age (/decade)**		0.87 (0.69, 1.09)	0.22
Cytogenetic risk			
Standard risk	209	1.0 (reference)	
High risk	136	1.64 (1.09, 2.49)	0.02
Unknown risk	16	1.54 (0.65, 3.63)	
**Disease status**			0.46
CR/sCR	75	1.0 (reference)	
VGPR	146	1.23 (0.69, 2.17)	
PR/NR/SD	140	1.43 (0.81, 2.53)	

**Table 4C:** Overall survival at 2-years post transplant.

Overall survival				
Melphalan day	N	2-year OS	95 CI%	p-value
−2	264	94%	90%–96%	0.76
−1	97	94%	84%–98%	

**Table 5A:** Univariate analyses of readmission rates by day of melphalan conditioning.

Factors	Day of melphalan conditioning		p
	Day −2	Day −1	
**N**	223	97	
**Hospital admission for melphalan (days)**			<0.01
Median (range), (IQR)	3 (2–51), (3–3)	2 (1–21), (2–2)	
**Hospital readmission within 30 days**	88 (39%)	55 (57%)	<0.01
**Duration of hospital readmission**			0.91
Median (range), (IQR)	6 (1–24), (4–8)	6 (1–33), (4–9)	

**Table 5B:** Multivariable logistic regression model of hospital readmission incidence by day of melphalan conditioning.

Factor	N	Odds ratio of readmission (95% C.I.)	p
Melphalan day			
−2	223	1.0 (reference)	
−1	97	1.91 (1.17, 3.11)	0.01
**Age (/decade)**		1.23 (0.93, 1.62)	0.15
**KPS**			0.05
90–100	227	1.0 (reference)	
60–80	93	1.65 (1.00, 2.70)	

Hospital readmissions within 30 days were significantly higher in the D-1 cohort (57%) vs. the D-2 cohort (39%) (p < 0.01) (**Table 5**). The most common reason for readmission was febrile neutropenia or infection. The median duration of hospital readmission was similar at 6 days in both cohorts D-2 (range 1–24 days) vs. D-1 (range 1–33 days). In a multivariable logistic regression model including age and KPS, D-1 melphalan had an odds ratio of 1.91 (95% CI 1.17–3.11; p = 0.01) for readmission within 30 days. KPS was also associated with readmission with patients who had a KPS of 60–80 having an odds ratio of 1.65 (95% CI 1.00–2.70; p = 0.05) compared with patients with a KPS of 90–100 (reference). The total number of admission days and clinic visits between day 0 and day 45 in the D-1 and D-2 cohort were the same (median 28, IQR 25–32).

## Discussion

Melphalan is the most frequently used conditioning agent for autologous stem cell transplant for multiple myeloma. Prior studies have provided mixed results regarding the optimal timing of melphalan administration for optimal disease outcomes and engraftment. In this cohort of 366 patients who underwent inpatient autologous stem cell transplantation, there was no significant difference in days to neutrophil or platelet engraftment.

We did find that administration of melphalan on D-1 was associated with an increased risk of readmission within 30 days. The patients in the D-1 group were slightly older than D-2, and we more had a low KPS (60–80) in the D-1 group (34% vs. 26%). In a logistic regression model of readmission, we found that KPS of 60–80 was significantly associated with a higher risk of readmission compared with KPS of 90–100. The older age and worse performance status in the D-1 groups contribute to the observed difference in readmission incidence. The average duration of hospital readmission did not differ between D-1 and D-2.

Importantly, PFS and OS at 2 years was similar in both D-1 and D-2 groups, suggesting that either dosing strategy is appropriate for disease control. This should be noted in instituting system-based practices for melphalan administration that will allow for shorter hospitalizations and decreased healthcare utilization and cost.

## Data availability statement

The raw data supporting the conclusions of this article will be made available by the authors, without undue reservation.

## Ethics statement

The studies involving humans were approved by University of Minnesota Institutional Review Board. The studies were conducted in accordance with the local legislation and institutional requirements. Written informed consent for participation was not required from the participants or the participants’ legal guardians/next of kin in accordance with the national legislation and institutional requirements.

## Author contributions

AM: Conceptualization, Data curation, Investigation, Writing – original draft, Writing – review & editing. RS: Formal analysis, Methodology, Writing – review & editing. FR: Data curation, Writing – review & editing. JL: Data curation, Writing – review & editing. MD: Data curation, Validation, Writing – original draft. SP: Writing – original draft. NE: Writing – review & editing. JR: Data curation, Writing – review & editing. KH: Data curation, Writing – review & editing. TD: Formal analysis, Methodology, Writing – review & editing. MJ: Writing – original draft. DW: Investigation, Methodology, Supervision, Writing – review & editing.
